# The effect of short-term mindfulness intervention on risk decision-making of final-year undergraduate students

**DOI:** 10.3389/fpsyt.2026.1904313

**Published:** 2026-09-30

**Authors:** Yani Duan, Nor Akmar Nordin, Siti Aisyah Binti Panatik, Chenyu Zhang, Xuefeng Gao, Yan Li

**Affiliations:** 1 Faculty of Social Sciences and Humanities, Universiti Teknologi Malaysia, Skudai, Malaysia; 2 School of Psychology and Mental Health, North China University of Science and Technology, Tangshan, China; 3 School of Business and Management Shanghai International Studies University, Shanghai, China

**Keywords:** final-year undergraduate students, mindfulness training, psychological intervention, risk decision-making, self-control resources

## Abstract

Mindfulness training holds unique value in enhancing decision-making, particularly under uncertainty. Against the backdrop of Chinese fresh graduates facing mounting employment pressure, widened wealth gaps, and a heightened tendency for high-risk decisions, this study draws on Risk Sensitivity Theory (RST) and the Self-Control Resource Model (SCM). Employing a 2×2 mixed experimental design, it explores the effect of a 4-week mindfulness training on final-year undergraduate students’ risk decision-making. Forty-four students were divided into a Mindfulness Group and a Control Group, with assessments conducted using uncertainty decision tasks and the FFMQ-20 scale. Results indicate the Mindfulness Group exhibited a significant reduction in high-risk decision probability and shorter decision reaction time. The findings suggest that short-term mindfulness training can suppress reliance on intuitive thinking and enhance prudent decision-making. These results are discussed within the theoretical frameworks of Risk Sensitivity Theory and the Self-Control Resource Model.

## Highlights

- 4-week mindfulness training reduces high-risk decisions in Chinese graduates.
- Mindfulness group shows faster decision reaction times than control.
- Training eases resource scarcity perception to improve decision-making.

## 1 Introduction

Risk decision-making, as a cognitive process through which individuals integrate probability estimation and outcome preferences in uncertain situations (1), presents a unique and pressing reality in the development of contemporary youth. With the expansion of higher education in China and the economic structural transformation in the post-pandemic era, recent graduates face decision-making difficulties regarding multiple development paths such as employment, entrepreneurship, and further education (2). Data from the Ministry of Education show that the scale of new graduates has exceeded ten million for four consecutive years from 2020 to 2023. In June 2023, the unemployment rate among youth aged 16–24 climbed to a historic peak of 21.3% (3). World Bank enterprise survey data indicate that the COVID-19 pandemic significantly reduced the survival probability of small and micro-enterprises and led to widespread firm closures, resulting in an estimated contraction of employment opportunities of around 10–15%, particularly in service-oriented and labor-intensive sectors (4). This supply-demand imbalance directly leads to the risk of poor decision-making. For example, the turnover rate of the class of 2020 within half a year after starting their jobs was significantly higher than before the pandemic (5), highlighting the continuous impact of risk decision-making biases on career trajectories.

From a macroeconomic perspective, between 2002 and 2022, China’s GDP grew at an average annual rate of 7.8%, but the Gini coefficient rose from 0.412 to 0.467 (3), reflecting the structural imbalance in the distribution of growth dividends. A national survey by Cao et al. (6) found that 78.3% of graduates perceive a mismatch between income and educational investment, with 63.5% tending to choose “high-return, high-risk” career paths.

This phenomenon aligns with the core assumption of Risk Sensitivity Theory (RST) - when individuals perceive a “resource gap” between their ideal state and actual situation, they systematically increase their risk preferences (7). More broadly, risk-based approaches emphasize the importance of integrating systematic risk assessment into decision processes under uncertainty (8). However, existing research mainly focuses on the direct impact of economic pressure on decision-making and lacks a systematic exploration of psychological intervention mechanisms (9), especially the potential role of mindfulness training in regulating cognitive resources and emotional processing.

Mindfulness refers to the psychological practice of consciously attending to present-moment experiences with a non-judgmental stance (10). Broad-spectrum evidence demonstrates that mindfulness can improve emotion regulation, mitigate stress responses, and boost executive-function performance (11, 12). These general cognitive-affective benefits lay a foundation for its potential influence on risky choice.

A growing body of behavioral studies has directly explored how mindfulness shapes risk-taking, uncertainty-based choice, impulsivity, and delay-discounting using standard economic-decision laboratory paradigms. Research adopting the Balloon Analogue Risk Task (BART) has shown brief mindfulness practice can moderate impulsive risky responding, though effect magnitudes vary across sample characteristics (13). Studies employing the Iowa Gambling Task (IGT) indicate that mindfulness-related training may improve advantageous decision-making under ambiguity by reducing perseverative loss-chasing tendencies (14). Delay-discounting paradigms further reveal that mindfulness interventions can alter temporal preference, lowering the degree to which individuals favor smaller-sooner rewards over larger-later payoffs, which is closely tied to real-world impulsive risk-taking (15). Nevertheless, the existing empirical base draws heavily on general-community participants or clinical populations; most relevant evidence is generated from de-contextualized laboratory gambling tasks. Few investigations have translated these laboratory findings to ecologically meaningful real-life choice contexts.

Existing research has predominantly focused on the mental health benefits of mindfulness, yet its underlying mechanisms for influencing risk decision-making remain insufficiently elucidated. Building on the Self-Control Resource Theory (Baumeister, 2007), decision-making behavior relies on limited cognitive resources (16), where resource depletion (Ego Depletion) prompts reliance on intuitive systems, subsequently increasing tendencies toward high-risk choices (17). Mindfulness training may mitigate ego depletion by reducing negative emotion-induced cognitive load (18) and enhancing impulse control capacities (19), thereby potentially strengthening deliberative decision-making. Furthermore, according to RST (7), perceptions of resource scarcity heighten propensity for risky strategies aimed at bridging ideal-reality gaps. Mindfulness might attenuate such perceptual biases, consequently diminishing non-rational risk-taking behaviors.

Based on Self-Control Resource Theory (SCM; 20), decision-making behavior relies on limited cognitive resources, and resource depletion leads individuals to depend on the intuitive system, increasing the tendency for high-risk choices (17). Mindfulness training can effectively alleviate self-control resource depletion and enhance prudent decision-making capabilities by reducing the consumption of cognitive resources by negative emotions (18) and strengthening inhibition control over immediate impulses (19, 21). Moreover, RST emphasizes that the perception of resource scarcity triggers activation of the β-adrenergic system, leading to decreased activation levels in the dorsolateral prefrontal cortex (dlPFC) and increased activity in the amygdala (22).

Mindfulness may inhibit this neurophysiological response by reducing the “perception of resource threat” and decrease irrational risk-taking behavior. In clinical populations, mindfulness training has been proven to enhance executive functions (23), and it has shown decision-improving effects in managerial contexts (24). However, its applicability in young graduates during this critical life transition period remains to be verified. The limitations of existing research are twofold: First, there is a lack of integrated analysis of the dual mechanisms (cognitive resource regulation and emotional threat perception) through which mindfulness affects risk decision-making; second, the dose-effect relationship of the intervention has not been fully considered (25).

Building upon the foregoing theoretical foundations, this study integrates the Self-Control Resource Model (SCM) and Risk Sensitivity Theory (RST) into a sequential explanatory framework rather than treating them as two independent parallel accounts. According to this sequential mechanism, mindfulness training firstly exerts its protective function on self-control resources: sustained attention and non-judgmental awareness mitigate automatic threat-triggered reactivity and replenish limited self-regulatory capacity depleted by job-hunting-related stress and uncertainty among final-year undergraduate students. The preserved self-control capacity further reshapes individuals’ risk appraisal processes, which can be interpreted from the perspective of RST. When sufficient self-control resources are available, individuals are less prone to activate a loss-framed resource-scarcity mindset that drives risk-seeking under perceived threat, and become capable of evaluating uncertain options in a more deliberate manner. To summarize the causal pathway: Self-Control Resource preservation→altered risk appraisal per RST→change in risk decision-making behavior.

Based on the foregoing causal pathway derived from the Self-Control Resource Model (SCM) and Risk Sensitivity Theory (RST), this study constructs a quantitative model for improving risk decision-making through mindfulness training, focusing on two core dimensions: risk choice probability (decision quality), and reaction time (decision efficiency). Specifically, a 4-week intervention period is selected based on the dose-effect curve analysis by Krusche et al. (25), aiming to reveal the possibility of changing risk decision-making patterns through psychological intervention. In graduates under employment pressure, mindfulness training aims to reduce excessive risk-taking caused by the “perception of resource threat,” helping them make more rational decisions in job searching and career planning, and reducing the adverse consequences of impulsive decision-making. Based on this, we proposed two hypotheses:

- H1: Compared with the Control Group (CG), the Mindfulness Group (MG) that received 4-week mindfulness training showed a significant decrease in the probability of high-risk choices in the uncertainty decision-making task (based on Risk Sensitivity Theory).
- H2: Compared with the CG, the MG had significantly shorter decision reaction times (based on the Self-Control Resource Model).

## 2 Methods

### 2.1 Participants

This study selects Chinese final year undergraduates as the research population. To determine the sample size needed to undertake this study, the researcher estimates the minimum sample size through a software application, G* Power. In this study, a study subject is an individual unit of analysis. Therefore, one undergraduate university student is considered as one individual. When using this approach to calculate sample size, the researcher chose the F tests family, using the ANOVA: Repeated measures, within-between interaction procedure to match the study’s 2 (group) × 2 (time) mixed ANOVA design. The settings of the program were set with anticipated effect size, *f* of 0.2, the desired alpha level of 0.05, number of groups = 2, number of measurements = 2, and the desired statistical power of 0.80. Based on the G* Power approach, the required sample size for the study is 42 individuals in the study. This shows that 42 subjects would provide 80% power (1 − *β* = 0.80) to detect the hypothesized group × time interaction at *α* = 0.05 (26–28), and the sample size of this study is similar to that of previous studies of the same type (29, 30).

This study recruited participants from North China University of Science and Technology. Invitation emails were distributed to all graduating undergraduate students, yielding an initial screening pool of 183 students. Thirteen individuals were excluded for failing pre-specified inclusion-exclusion criteria, leaving 170 eligible participants who completed the Uncertainty Decision-Making Task (UDP) as the screening measure. Following the classic extreme-group selection rule (31, 32), participants with UDP scores in the top 27% of the sample distribution were short-listed. From this subset, 46 candidates with a 1:1 male-to-female ratio were selected via this rank-based top-N approach for potential formal-study enrollment. Two candidates withdrew before the formal experiment due to scheduling conflicts that precluded full participation in the multi-week intervention. The final sample comprised 44 participants (22 males, 22 females). All participants provided written informed consent. Eligibility criteria were: undergraduate graduates aged 18-26years, right-handed, normal or corrected-to-normal vision, no history of chronic or mental illness, no prior mindfulness training, non-Psychology/Economics majors, and no urgent financial needs. Participants with urgent financial needs were excluded for ethical-safety considerations given the psychological burden of the risk-decision task and multi-week study commitment; this may limit our sample to individuals with low-to-moderate perceived resource scarcity. After final short-listing of the 44 participants, computer-generated simple randomization was used to assign participants to the Mindfulness Group (MG, n=22) or no-intervention Control Group (CG, n=22). The random sequence was generated and implemented by a research assistant independent of data collection and outcome assessment. No additional dropout occurred post-randomization, and no valid behavioral trial data were missing across pre-test and post-test. Behavioral-task administrators were not blinded to group allocation; quantitative analyses were conducted on de-identified datasets stripped of group labels to minimize analytic bias. Participant demographics: age *M*=22.3±1.5years (MG: 22.1±1.4years; CG: 22.5±1.6years); majors: liberal arts18 (40.9%), science16 (36.4%), engineering10 (22.7%); monthly living expenses *M*=1850±320yuan. No significant between-group differences were observed for age, gender, or major distribution (ps>0.05).

### 2.2 Research design

A mixed experimental design of 2(group: Mindfulness Group [MG], Control Group [CG]) × 2(measurement time: pre-test, post-test) was adopted, in which different tasks were between-subject variables and measurement time were within-subject variables. The MG received the mindfulness training (MT), while the CG received no training. The purpose of using a control group is to quantify the effect of random and uncontrollable variables. Therefore, in this research, the results of the MG were compared with those of the CG.

The experiment included pretests (the first day) and post-tests (four weeks later). In addition, for mindfulness training, in traditional clinical practice, most researchers use 8 weeks as the “standard” length of intervention (33). However, in recent years, studies have shown that 8 weeks of training is not the lowest “dose” to produce an effect, and studies have shown that the treatment effect is not strongly correlated with the duration of the intervention program or the number of interventions (34). Studies have shown that a 3-week short-term mindfulness meditation training can improve the general quality of life of patients undergoing coronary reangioplasty and significantly reduce the symptoms of tension chronic headache (35). Five weeks of mindfulness training can effectively improve individuals’ emotional regulation ability and psychological well-being (36), and help students effectively cope with stress (37). These studies suggest that short-term mindfulness interventions of 3–6 weeks can also have positive effects in different ways. Meanwhile, Westbrook et al. (38) found that individual executive function could be significantly improved through short-term mindfulness training. The results of a mindfulness training for college students showed that after four-days continuous mindfulness training, the individual’s attention and executive function were significantly improved. Therefore, the researchers assessed the participants’ probability and reaction time of uncertainty decision-making before and after the training and throughout the four weeks.

Additionally, subject fees and gifts were given to all participants as an incentive for full participants on all days. The intervention program consisted of ten core mindfulness practices, including the raisin eating exercise, body scan, and mindfulness stretching. Detailed protocols and instructional steps for each practice are provided in **Supplementary Appendix A2**.

### 2.3 Instrumentation

#### 2.3.1 Questionnaire

Mindfulness Attention Perception Scale (FFMQ-20) is a Chinese simplified version of the Mindfulness Awareness Scale developed by Hou et al. (39) on the basis of the original FFMQ scale and applicable to the Chinese cultural background. On the premise of retaining the basic dimension structure of the original scale, the author retains four items with the highest correlation coefficient with the original scale in each dimension. The FFMQ-20 scale was used to assess changes in participants’ mindfulness levels before and after the intervention, serving as a manipulation check to verify the effectiveness of mindfulness training. The original scale comprised four dimensions with four items each. Based on confirmatory factor analysis, one item with low factor loading was removed, resulting in a final version consisting of 15 items across four dimensions. The overall reliability of the revised scale was high (Cronbach’s *α* = 0.96), explaining 91.3% of the variance of the original scale. The four dimensions assessed were (1) attention awareness, (2) emotional awareness, (3) present-moment focus, and (4) goal-oriented automaticity. Sample items included statements such as “I may be managing some emotion, but I do not notice it until some time later,” “I have trouble focusing on what is happening in the present moment,” and “I am too focused on pursuing goals and ignore the process.” Participants rated each item on a six-point Likert scale (1 = never, 6 = always), reverse-scored items were recoded prior to summation such that higher total scores indicate higher levels of mindfulness attention, consistent with the scoring convention used in the results.

#### 2.3.2 Performance test

We use the Uncertainty Decision-Making Task as the risk decision-making task. The stimuli are presented to the participants through a 21-inch Dell HD CRT color display, which is centered 80 cm away from the subjects with a viewing angle of 3.5°. The stimuli are presented by E-Prime 2.0 software, while the responses of the subjects in the task are recorded using a computer keyboard. Two options are presented on the left and right side of the screen: “100% get $10” and “20% get $20” (i.e., low uncertainty, low return and relatively high uncertainty, high return), which are presented in a circle with the percentage associated with each option and an explanation. In each trial, participants chose between a certain option (100% chance of receiving 10 units) and an uncertain option with varying probability–return combinations. The uncertain options included three probability levels (0.20, 0.30, 0.50) paired with proportionally higher returns (20, 30, and 50 units, respectively).

For hypothesis testing, risk preference was operationalized dichotomously at the choice level. Specifically, selections of the uncertain option—regardless of probability level—were coded as high-risk choices (coded as 1), whereas selections of the certain option were coded as low-risk choices (coded as 0). The probability of high-risk choices was calculated as the proportion of trials in which the participant selected the uncertain option relative to the total number of trials (30). Reaction time analyses included only valid trials (300 ms < RT < 5000 ms), and the mean reaction time across valid trials was used as the final indicator. This experimental procedure is based on previous research (40, 41). The combination of probability and payoff, the left and right positions of uncertainty-free and uncertainty presentations are all at random, and the decision and money accumulation results are presented on the last screen of each trial round, and the participant’s reward is the final cumulative amount. For each trial, the fixation point of 1000ms is presented first; then they are presented with an experimental stimulus until they respond; then a 1500ms blank screen is presented; finally, the result feedback and cumulative amount of the trial last 1000ms (**Figure 1**).

**Figure 1:**
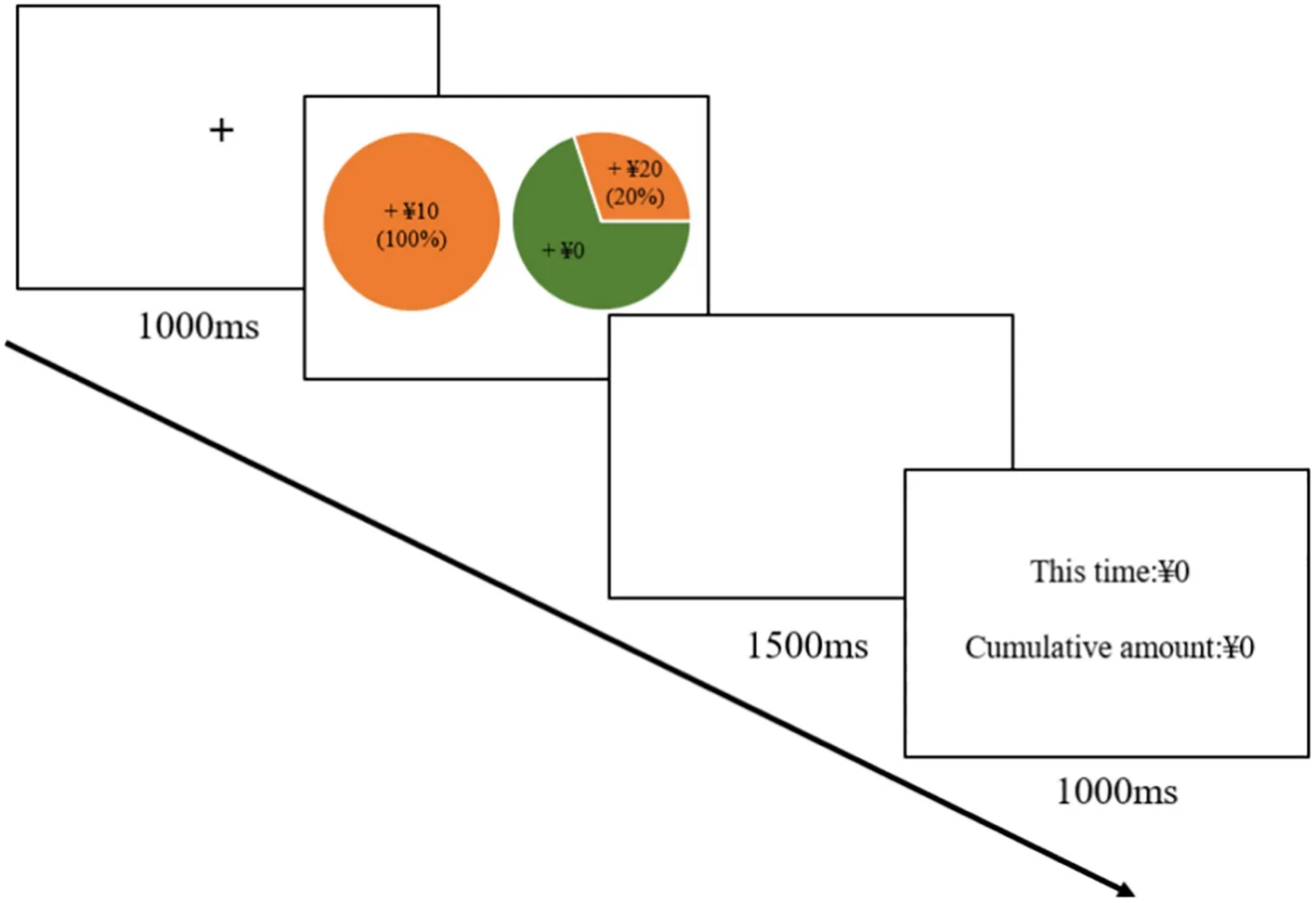
Time process of a single task trial.

#### 2.3.3 Mindfulness training

This study used a 4-week randomized controlled intervention design for mindfulness training to test whether 4-week mindfulness training can promote the reduction of uncertainty decision-making behaviors of non-clinical subjects. According to the content of 8-week Mindfulness-based stress reduction course, 8-week Mindfulness-based Cognitive Therapy (MBCT) course (33), we designed a 4-week Mindfulness-based self-growth education training course. A 4-week duration was selected based on the dose-effect curve reported by Krusche et al. (25), which indicates that measurable improvements in attentional control and emotion regulation typically emerge from around 3–4 weeks of regular mindfulness practice, with diminishing marginal benefit, relative to increased attrition risk, beyond this range; this duration also represented the longest intervention window feasible within the final semester before participants’ graduation and the onset of intensive job-search activity, while remaining consistent with the dosage used in comparable brief mindfulness interventions with student populations (e.g., 37). The mindfulness course was led by 2 psychological counselors holding Level-2 MBCT (Mindfulness-Based Cognitive Therapy) certificates, both with more than 5 years of mindfulness intervention experience. They received unified training before the intervention to ensure procedural consistency. The course plan is shown in **Supplementary Appendix A1**. In terms of course content and structure, compared with the standard 8-week mindfulness meditation course, the 4-week mindfulness training scheme in this study mainly lacks the content of a full-day “Silence” meditation practice and “Mindfulness yoga”. Other course contents (including homework) and course duration are not significantly different from the standard 8-week mindfulness meditation practice. The duration of the mindfulness meditation intervention training course is 4 consecutive weeks, about 2-2.5 hours of intensively guided practice once a week. As recommended by Siegling et al. (42), each of our intensive instruction sessions (except the first) follows the following sequence structure: “Start practicing,” “Feedback (including feedback on homework practice),” “Psychoeducation,” “Re-practice,” “Re-feedback,” “Assign homework,” “End”. At the same time, the participants are given corresponding learning topic materials and homework materials (including homework practice record sheet) in each class, and are required to actively complete the corresponding formal and informal mindfulness meditation exercises after class and make the corresponding records.

### 2.4 Procedure

The participants in the MG were told that the experiment was divided into three parts: the first part took place on the same day, the second part lasted 4 weeks, and the third took place four weeks later. Participants in the CG did not receive any treatment or information related to mindfulness training. There was also no requirement for participants during the pretest and post-test period. They performed the pre-uncertainty-decision making task at baseline and again on the last day of the experiment.

The study adopted a pre-test-post-test control group design, with the procedure for both groups shown in **Table 1**; **Figure 2**.

**Table 1:** Research procedures for two groups.

Stage	Mindfulness group (MG)	Control group (CG)
Pre-test (T1)	• Completed the Mindfulness Attention Perception Scale (FFMQ-20); • Performed the uncertainty decision-making task	Performed the uncertainty decision-making task only
Intervention (Weeks 1-4)	Session of guided mindfulness training (2-2.5 hours/week) + daily after-class exercises (e.g., body scan, mindful eating)	No intervention; no access to mindfulness-related information
Post-test (T2)	• Re-completed the FFMQ-20; • Performed the uncertainty decision-making task again	Performed the uncertainty decision-making task again

**Figure 2:**
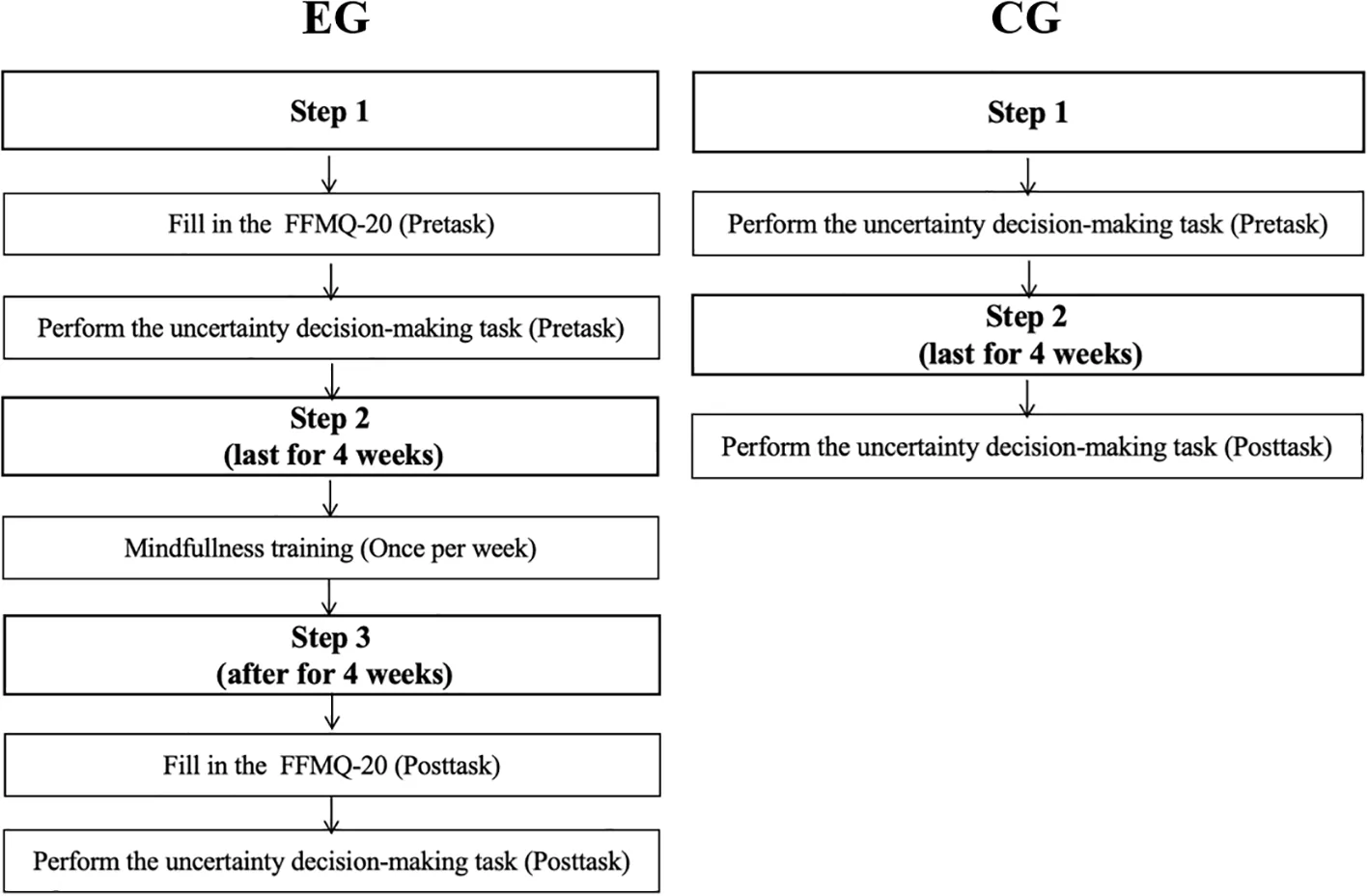
Flowchart of research procedures for two groups.

## 3 Results

All statistical analyses were performed using SPSS 26.0. Prior to analysis, assumption checks for mixed ANOVA were systematically conducted. The Shapiro–Wilk test confirmed normality of residuals, Levene’s test verified homogeneity of variance, and Mauchly’s test supported the sphericity assumption for all repeated-measure comparisons. No severe assumption violations were observed. Missing data were minimal (< 1%) and handled via full information maximum likelihood (FIML) estimation. For behavioral task data, reaction-time trials with anticipatory responses (< 300ms) or timeout failures (> 5000ms) were excluded; in total, 2.14% of all trials were removed, consistent with standard behavioral data filtering criteria. All post-hoc simple-effect comparisons were performed with Bonferroni correction to control TypeI error inflation, with the adjusted alpha level set at 0.05. Descriptive statistics for FFMQ-20 mindfulness scores, high-risk choice probability, and decision reaction time across the two groups at pre-test and post-test are presented in **Table 2**. All data are reported as mean±standard deviation (*M*±SD), together with 95% confidence intervals (95%CIs) and standardized effect sizes.

**Table 2:** Descriptive statistics of variables for two groups (*M* ± SD).

Variable	EG - Pre-test	EG - Post-test	CG - Pre-test	CG- Post-test
FFMQ-20 Score	45.2 ± 6.8	58.7 ± 7.2	44.8 ± 7.1	45.3 ± 6.9
High-Risk Choice Probability	0.47 ± 0.20	0.33 ± 0.15	0.46 ± 0.18	0.47 ± 0.18
Decision Reaction Time (ms)	1890.97 ± 762.2	1270.05 ± 498.5	1405.23 ± 762.22	1399.27 ± 498.25

### 3.1 Results for reaction time

A repeated-measures ANOVA revealed no significant main effect of group, *F*(1,42) = 2.13, *p* = 0.151, *η_p_^2^* = 0.048, 95% *CI* [0.000, 0.187]. The main effect of time (pre- vs. post-intervention) was significant, *F*(1,42) = 4.59, *p* = 0.038, *η_p_^2^* = 0.094, 95% *CI* [0.004, 0.233], with longer reaction times pre-intervention (*M* = 1648.10, *SD* = 764.78) compared to post-intervention (*M* = 1334.66, *SD* = 500.22). A significant interaction effect emerged, *F*(1,42) = 4.41, *p* = 0.041, *η_p_^2^* = 0.091, 95% *CI* [0.003, 0.229]. Bonferroni-corrected simple-effects analyses revealed that the MG showed significantly reduced reaction times from pre- (*M* = 1890.97, *SD* = 762.2) to post-intervention (*M* = 1270.05, *SD* = 498.5), *p* < 0.05, *Cohen’s d* = 0.95, 95% *CI* [0.38, 1.51] (**Figure 3**); while the CG exhibited no significant change (pre: *M* = 1405.23, *SD* = 762.22; post: *M* = 1399.27, *SD* = 498.25), *p* = 0.947, *Cohen’s d* = 0.01, 95% *CI* [−0.41, 0.43].

**Figure 3:**
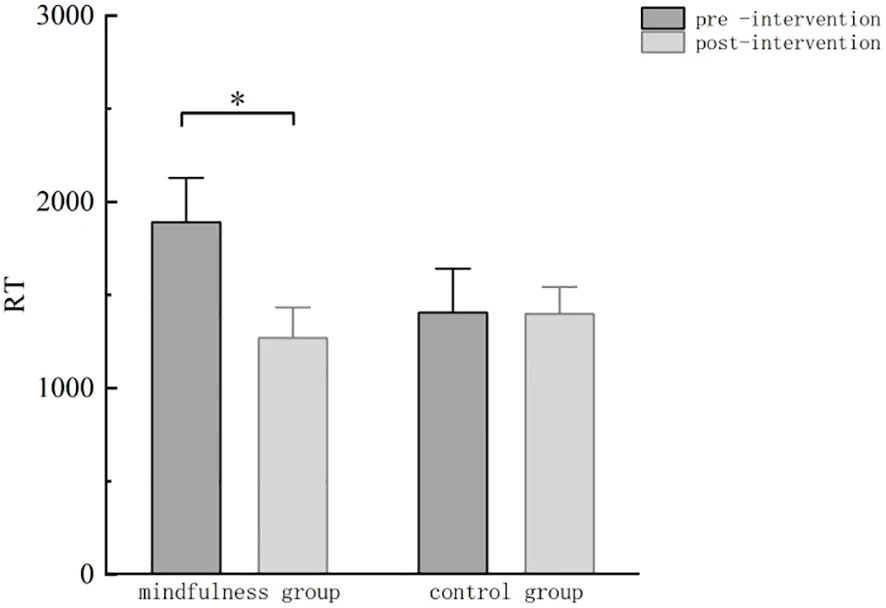
Comparison of decision reaction time between MG and CG *p < 0.05.

For non-risk decision reactions, a mixed ANOVA revealed no significant main effect of group, *F*(1,42) = 1.45, *p* = 0.235, *η_p_^2^* = 0.033. The main effect of time was marginally significant, *F*(1,42) = 3.18, *p* = 0.082, *η_p_^2^* = 0.067, reflecting slightly faster responses at post-test, with slower reaction times pre-intervention (*M* = 1511.88, *SD* = 598.33) than post-intervention (*M* = 1314.20, *SD* = 564.37). The interaction effect was non-significant, *F*(1,42) = 1.81, *p* = 0.185, *η_p_^2^* = 0.041, indicating that temporal changes in general decision speed were comparable across groups.

To clarify the basic characteristics of variables and their interrelationships, **Table 2** presents the descriptive statistics (Mean ± SD) of FFMQ-20 scores (mindfulness level), high-risk choice probability (decision quality), and reaction time (decision efficiency) for both groups at pre-test and post-test. **Table 3** presents the Pearson correlation coefficients among the three core variables.

**Table 3:** Pearson correlation matrix among variables.

Variable	Mindfulness level	High-risk choice probability	Decision reaction time
Mindfulness Level (FFMQ-20)	1	-0.42**	-0.38*
High-Risk Choice Probability	-0.42**	1	0.29*
Decision Reaction Time	-0.38*	0.29*	1

*Correlations are based on post-test measurements.*p < 0.05, **p < 0.01.

As shown in **Table 2**, the MG exhibited a significant increase in FFMQ-20 scores (pre-test: 45.2 ± 6.8; post-test: 58.7 ± 7.2), alongside decreases in high-risk choice probability (pre-test: 0.47 ± 0.20; post-test: 0.33 ± 0.15) and reaction time (pre-test: 1890.97 ± 762.2 ms; post-test: 1270.05 ± 498.5 ms) after the intervention. In contrast, the CG showed no noticeable changes in any variables.

As shown in **Table 3**, mindfulness level (FFMQ-20 score) was significantly negatively correlated with high-risk choice probability (*r* = -0.42, *p* < 0.01) and reaction time (*r* = -0.38, *p* < 0.05), indicating that higher mindfulness was associated with more prudent decision-making and faster decision efficiency. A positive correlation was observed between high-risk choice probability and reaction time (*r* = 0.29, *p* < 0.05), suggesting that impulsive high-risk decisions may not be accompanied by efficient information processing.

### 3.2 Results for risk decision probability

A mixed ANOVA on risk decision probability revealed no significant main effect of group, *F*(1,42) = 2.81, *p* = 0.101, *η_p_^2^* = 0.063. The main effect of time was significant, *F*(1,42) = 4.34, *p* = 0.043, *η_p_^2^* = 0.09, 95% *CI* [0.002, 0.228], with higher risk choice frequency pre-intervention (*M* = 0.47, *SD* = 0.18) than post-intervention (*M* = 0.40, *SD* = 0.17). A significant interaction effect was observed, *F*(1,42) = 4.87, *p* = 0.033, *η_p_^2^* = 0.10, 95% *CI* [0.006, 0.245]. Bonferroni-corrected simple-effect tests confirmed that the MG reduced risk choices from pre- (*M* = 0.47, *SD* = 0.20) to post-intervention (*M* = 0.33, *SD* = 0.15), *p* < 0.05, *Cohen’s d* = 0.78, 95% *CI* [0.22, 1.33], while the CG showed no significant change (pre: *M* = 0.46, *SD* = 0.18; post: *M* = 0.47, *SD* = 0.18), *p* = 0.821, *Cohen’s d* = 0.06, 95% *CI* [−0.36, 0.48]. As shown in **Figure 4**, the mindfulness group exhibited a significant decrease in high-risk choice probability from pre-test to post-test, whereas the control group showed no significant change.

**Figure 4:**
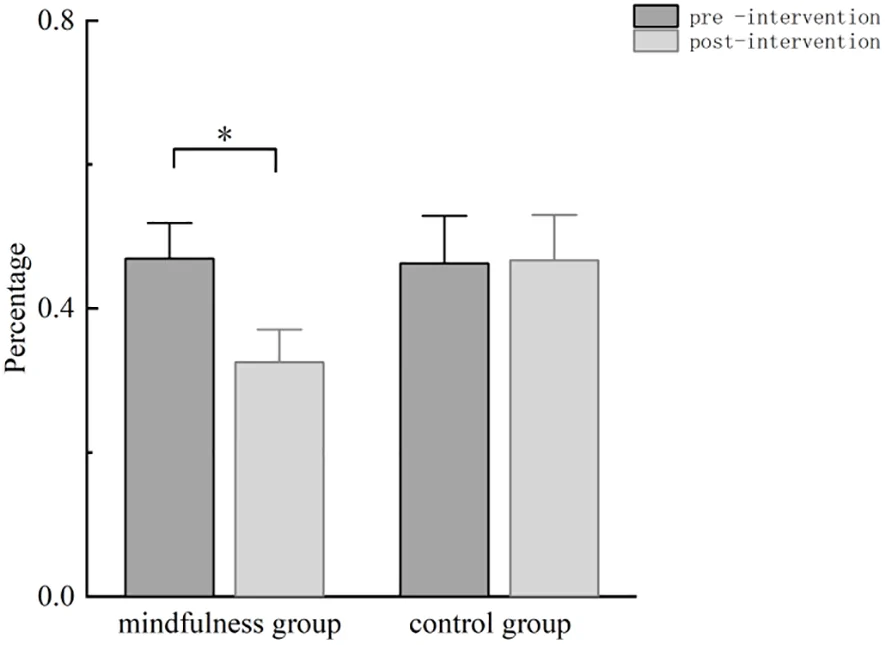
Comparison of high-risk choice probability between MG and CG, *p < 0.05.

To provide a more nuanced analysis, we disaggregated the overall risk choice probability into its three constituent probability-payoff conditions. A 2 (Group) × 2 (Time) × 3 (Condition: 0.20/20, 0.30/30, 0.50/50) mixed-design ANOVA revealed a significant main effect of Condition, *F*(2, 84) = 34.52, *p* <.001, *η_p_^2^* = 0.45, Bonferroni-corrected post-hoc tests showed that the probability of choosing the 0.50/50 option (*M* = 0.68, *SD* = 0.21) was significantly higher than that for the 0.30/30 option (*M* = 0.42, *SD* = 0.19) and the 0.20/20 option (*M* = 0.19, *SD* = 0.14), ps <.001 (**Table 4**), indicating that participants were more likely to choose options with higher expected payoffs. Critically, the three-way interaction was significant, *F*(2, 84) = 3.89, *p* = .024, *η_p_^2^* = 0.085. Simple effect analyses found that the mindfulness group significantly reduced their choice probability for the 0.50/50 option from pre- to post-intervention, while the control group showed no significant change (*p* >.05). No significant effects were found for the 0.20/20 and 0.30/30 conditions (ps >.10). 

**Table 4:** Probability of choosing each uncertain option by group and time (*M* ± SD).

Condition (Probability/Payoff)	EG - Pre-test	EG - Post-test	CG - Pre-test	CG - Post-test
0.20/20	0.20 ± 0.15	0.18 ± 0.12	0.19 ± 0.14	0.21 ± 0.13
0.30/30	0.43 ± 0.18	0.41 ± 0.16	0.42 ± 0.17	0.43 ± 0.18
0.50/50	0.72 ± 0.18	0.55 ± 0.16	0.67 ± 0.19	0.65 ± 0.20

A mixed-effects logistic regression model predicting trial-by-trial choice (uncertain vs. certain) further elucidated this pattern. The significant Group × Time × Expected Value (*EV*) interaction (*B* = -0.18, *SE* = 0.08, *Wald χ²* = 5.06, *p* = .024) indicated that the mindfulness training reduced participants’ sensitivity to EV. While the control group showed no change in *EV* sensitivity over time, the mindfulness group exhibited a significant decrease (*B* = -0.21, *SE* = 0.10, *p* = .035). This suggests that post-intervention, the mindfulness group relied less on the objective economic value when making decisions, which aligns with a shift from a purely rational-economic calculation to a more deliberate and cautious evaluation, particularly when faced with highly attractive but risky options.

## 4 Discussion

This study employed a 4-week mindfulness training intervention to examine its dual mechanisms—emotion regulation and cognitive resource preservation—in modulating risk decision-making behaviors among Chinese college graduates (43). Results aligned with the Risk Sensitivity Theory (RST) and Self-Control Resource Model (SCM). Relative to the CG, participants in the MG was associated with lower perceived resource scarcity and reduced reliance on intuitive decision-processing, correlating with improvements in both decision-quality indicators and decision-making efficiency.

THypothesis 1 was fully supported: the experimental group’s high-risk choice probability decreased by 14% after four weeks of mindfulness training, while the control group showed no significant change. This finding is consistent with the core prediction of Resource Scarcity Theory (RST; 7), which posits that individuals’ risk-taking behaviors are shaped by their subjective perceptions of resource adequacy rather than objective resource levels. From this perspective, mindfulness training may have reduced participants’ perceived resource gaps—specifically, the discrepancy between ideal and attainable employment outcomes—by fostering non-judgmental awareness and emotional regulation. As a result, participants were less likely to adopt high-risk strategies as a compensatory response to perceived scarcity. The observed improvement in decision-making may also reflect enhanced self-regulatory capacity. By increasing individuals’ awareness of their internal states and strengthening their perceived ability to regulate behavior in accordance with longer-term goals, mindfulness may support more effective goal-directed behavior. This interpretation is consistent with the role of self-efficacy in human agency and behavioral self-regulation (44).

Within the context of graduate career decision-making, this psychological mechanism may translate into a reduced tendency to overestimate employment-related resource scarcity, thereby decreasing impulsive preferences for high-risk options such as unstable entrepreneurship or precarious employment. Importantly, this interpretation does not imply a reduction in motivation, but rather a shift toward more calibrated and deliberate decision strategies under perceived employment pressure. For graduates facing employment pressure, mindfulness training may reduce the overestimation of “employment resource scarcity” through non-judgmental awareness, thereby reducing impulsive choices such as “blindly pursuing high-risk entrepreneurship” or “accepting unstable jobs”. Hypothesis 2 was fully supported: the MG’s decision reaction time was shortened by approximately 620ms, while the CG showed no significant change. This is consistent with SCM (20): participants in the MG may reduce cognitive-resource depletion produced by negative emotions (e.g., job-hunting-related anxiety), which could foster more efficient systematic decision-processing. For example, the MG showed faster information processing when evaluating “stable low-salary jobs” and “unstable high-salary jobs”, which is related to enhanced attentional control (38).

It is worth noting that, for both reaction time and risk-choice probability, the main effect of group was non-significant, whereas the group × time interaction was significant. This pattern is consistent with our theoretical framework rather than undermining it: at baseline, the MG and CG were drawn from the same population and had not yet been exposed to differential training, so no baseline group difference was expected. A significant interaction alongside a non-significant main effect of group therefore indicates that mindfulness training altered participants’ risk decision-making over time, rather than reflecting a pre-existing, trait-like difference between groups. This is in line with the state-dependent framing of both Risk Sensitivity Theory and the Self-Control Resource Model, which conceptualize perceived resource scarcity and self-control capacity as dynamic states rather than stable traits; our interaction-driven pattern of results should accordingly be interpreted as evidence of a state-level change over the intervention period, and not as evidence of a stable shift in participants’ underlying risk disposition.

We also note that reaction time, while informative, is only an indirect proxy for decision efficiency: faster post-intervention responses are also consistent with alternative explanations such as increased impulsivity, reduced task engagement, or a speed-accuracy trade-off, rather than exclusively reflecting improved self-regulatory efficiency. Two aspects of our results speak against a simple speed-accuracy trade-off account, however: the MG’s reduction in reaction time (Hypothesis 2) was accompanied by a corresponding decrease, rather than an increase, in high-risk choice probability (Hypothesis 1), suggesting that participants were responding faster while also making more selective, lower-risk choices, rather than trading accuracy for speed. Nonetheless, because this study did not include a manipulation check for self-control resource depletion (e.g., a Stroop task or a validated state self-control scale), the attribution of the reaction-time findings to reduced ego depletion under the Self-Control Resource Model should be regarded as a plausible, theoretically motivated interpretation rather than a directly confirmed mechanism. Future research should incorporate such manipulation checks, together with trial-level accuracy and choice-consistency measures, to more rigorously test whether mindfulness-related speed changes reflect genuine gains in self-regulatory efficiency.

Universities should incorporate brief mindfulness modules (e.g., body scans, breathing exercises) into career counseling to foster rational decision-making under pressure (e.g., job interviews). Standardizing mindfulness as part of employment anxiety relief programs could mitigate high-risk behaviors (e.g., online scams) among youth. Based on the core finding that “4-week mindfulness training improves graduates’ risk decision-making”, practical recommendations are focused on university scenarios: First, integrate mindfulness modules into graduation-season career counseling: Universities can add 15-minute daily mindfulness exercises (e.g., body scans, mindful breathing) to career guidance courses, helping graduates cope with decision anxiety (e.g., anxiety about “delayed job offers”) and avoid impulsive choices. Then, targeted intervention for high-risk groups: For graduates with high resource scarcity perception (e.g., students from low-income families), add scarcity perception adjustment modules (e.g., cognitive reappraisal of “employment resources”) to the intervention program to enhance the intervention effect.

In addition, our findings provide a refined understanding of how mindfulness modulates risk-taking. While the initial global analysis (Hypothesis 1) suggested a broad reduction in high-risk choices, the disaggregated analysis reveals that this effect is primarily driven by a significant decrease in selecting the most lucrative, yet highly uncertain, option (0.50/50). This is a crucial nuance. It suggests that mindfulness does not induce a global, indiscriminate shift toward risk aversion. Instead, it appears to specifically temper the allure of decisions with a potentially high payoff, reducing impulsive choice toward options that are economically tempting but inherently volatile.

Furthermore, the results of the logistic regression analysis, showing a decreased sensitivity to Expected Value (*EV*) in the mindfulness group post-intervention, offer a compelling interpretation. This pattern aligns with the tenets of Dual Process Theory (45). It implies that mindfulness training may not necessarily enhance System 2 (deliberative, rational) processing in a way that makes decisions more economically optimal. Rather, it appears to dampen the influence of System 1 (intuitive, affective) processing, which is often driven by the prospect of a large reward (the “affective” component of the 0.50/50 option). By reducing this intuitive pull, participants are less swayed by the potential “big win” and may engage in a more balanced, cautious evaluation, leading to a lower probability of choosing the 0.50/50 option. This interpretation is consistent with the Self-Control Resource Model, suggesting that preserved cognitive resources from mindfulness training allow individuals to better inhibit the automatic impulse to chase high rewards, resulting in more prudent, but not necessarily more rational in an economic sense, choices.

The single-university sample limited generalizability. Future research should diversify samples across regions and disciplines. In addition, participants reporting urgent financial needs were excluded from the study; because Risk Sensitivity Theory centers on perceived resource scarcity, this exclusion may have removed individuals most relevant to the theoretical framework and likely restricted the sample to a low-to-moderate range of resource scarcity, which may in turn have restricted the observed range of risk-taking behavior and constrains the generalizability of our findings to more financially precarious populations.

Behavioral and self-report data were supplemented by theoretical inferences but lacked neuroimaging (46). Although this study did not directly measure the fluctuation of self-control resources, the results align with the Self-Control Resource Model. It is plausible that mindfulness training helps restore depleted cognitive resources, allowing graduates to resist impulsive, high-risk options in favor of more rational long-term gains. Future research could explicitly measure these mediating variables to validate this mechanism (47). Similarly, we did not formally compare the 4-week intervention duration used here against shorter or longer alternatives; while our choice was grounded in the dose-effect literature (25) and practical constraints of the academic calendar, a dose-comparison design would be needed to establish the optimal training duration for this population and outcome. Additionally, the study did not control for participants’ prior mindfulness experience, which may have influenced outcomes. Finally, cultural factors (e.g., collectivism-driven career expectations) warrant exploration in future cross-cultural comparisons.

Importantly, the present study utilized a no-contact wait-list-style control condition. The mindfulness-intervention group received regular guided group sessions, homework assignments, instructor contact, and structured group activities, whereas the control group received no comparable structured activity. Consequently, the observed group-by-time effects cannot definitively be attributed to mindfulness-specific content alone; outcomes could stem from non-specific factors including instructor attention, participant expectancy effects, group interaction processes, general relaxation, or repeated-task exposure. Without an active-control condition (for example, a health-education or relaxation-matched program equated for contact time, group format, and homework load), the study design cannot disentangle genuine mindfulness-specific effects from broad intervention-related or social-attention effects. Adopting rigorously-matched active-control groups represents an important priority for follow-up investigations aiming to isolate the unique effects of mindfulness content.

## 5 Conclusion

This study finds that a 4-week mindfulness training significantly reduces high-risk decision-making and reaction times among Chinese graduates. By fostering present-moment awareness, mindfulness helps individuals shift from impulsive, intuitive processing to more deliberate, rational decision-making. These results align with the Self-Control Resource Model, suggesting that mindfulness may buffer the cognitive depletion caused by employment pressure. Practically, mindfulness serves as a valuable psychological tool for universities to help graduates navigate the job market more prudently. Future research should explicitly measure self-control resources to further validate these underlying mechanisms.

## Data Availability

The raw data supporting the conclusions of this article will be made available by the authors, without undue reservation.

## Supplementary material

Supplementary Appendix A1 4-week mindfulness training scheme.

Supplementary Appendix A2 Mindfulness intervention protocols.
