# Supplementary material for: The effect of short-term mindfulness intervention on risk decision-making of final-year undergraduate students
Source: Front Psychiatry. 2026 Sep 30;17:1904313. doi: 10.3389/fpsyt.2026.1904313 (PMC13664286; doi:10.3389/fpsyt.2026.1904313)
Supplement: Supplementary file 1 [file SupplementaryFile1.pdf]

APPENDIX

Appendix 1 4-week mindfulness training scheme

Week	Mindfulness education activities	Homework
1	● **Theme: Introduction to the course and mindful awareness practice of somatosensory**<br>● **Purpose:**<br>To introduce the relevant requirements of the course and set confidentiality rules. To understand the three basic modes of mental activity: "automatic navigation mode", "pain avoidance mode" and "goal-action mode". To understand the meaning of mindfulness through body awareness practice.<br>● **Activities/Projects:**<br>(1) Course introduction; introduce yourself and share your motivation. (30 minutes)<br>(2) Psychological education of general modes of mindfulness and mental activities. (20 minutes)<br>(3) The "Raisin Eating" exercise . (15 minutes)<br>(4) Feedback and discussion of the "Raisin Eating exercise" activity. (5 minutes)<br>(5)"Body Scanning" exercise . (35 minutes)<br>(6) Feedback and discussion of "Body Scanning exercise". (5 minutes)<br>(7) Assign homework and distribute homework related materials.	(1) "Body Scan" Practice, 6 days a week<br>(2) Mindfulness practices in life like mindful eating, mindful bath, mindful self slap,... At least 1 item per day.<br>(3) Read "Clear Mind - A Metaphor for Mindfulness"

	(8) End the course	
2	● **Theme**: Mindfulness awareness practice for thoughts and feelings<br>● **Purpose**:<br>The understanding and practice of "ideas are not equal to facts" and "cognitive de-integration".<br>● **Activities/Projects:**<br>(1) Body Scan exercise (15 minutes).<br>(2) Practice feedback and homework feedback and review (15 minutes).<br>(3) Meditation practice and discussion (45 minutes).<br>(4) Mindfulness stretching + rest (10 minutes).<br>(5) Psychological education about the nature and relationship between emotions and thoughts<br>(" Street Encounter Exercise "(Teasdale, J., 2017)," Three-step dissociation Exercise "(15 minutes).<br>(6) "Idea Meditation Practice" (20 minutes).<br>(7) Homework assignment (5 minutes).<br>(8) End the course.	(1) "Body Scan" exercise, first 3 days; "Idea Meditation Practice" 3 days later<br>(2) River rafting practice.<br>(3) Mindfulness practices in life, like mindful eating, mindful bath, mindful self slap,... At least 1 item per day.
3	**Theme: Non-avoidance/Self-acceptance of experience**<br>**Purpose:**<br>Understanding the meaning of non-avoidance/acceptance of experience through psychological education and practice.	(1) 10 minutes of mindful breathing exercises, 6 days a week.<br>(2) Listening to Zen Exercises 23.<br>(3) Mountain Meditation

	**Activities/Projects:**<br>(1) Sitting meditation practice (45 minutes).<br>(2) Exercise feedback and homework feedback and review (5 minutes)<br>(3) Psychological education of experience avoidance/acceptance (5 minutes).<br>(4) Mindfulness stretching + rest (10 minutes).<br>(5) "Mindfulness Meditation on Difficulties" practice (30 minutes).<br>(6) Steps of emotional acceptance (5 minutes).<br>(7) The "three-minute breathing space" exercise (5 minutes).<br>(8) Assign homework and end the class (5 minutes).	Practice<br>（4）Mindfulness practices in life, like mindful eating, mindful bath, mindful self slap,... At least 1 item per day.<br>(5) Read "acceptance" metaphorical educational materials -The Guest Room (Rumi), Mindfulness-based Cognitive Therapy for Depression (Second Edition), p286
4	● **Theme**：**Live fully at the present**<br>● **Purpose**：Experience "being fully present and experiencing the process of being aware of Myself"<br>● **Activities / Projects**：<br>(1）Compassion Body Scan (20 minutes).<br>(2) Family practice review (10 minutes).<br>(3) Mindfulness stretching + rest (10 minutes).<br>(4) Introduce simple ways to get in touch with the present (5 minutes).<br>(5) "Metaphor of the Garden" meditation practice (15 minutes).<br>(6) Review, discussion and summary of the whole course (15 minutes).<br>(7) Issue questionnaires and finish the course	(1)Encourage/suggest mindfulness for everyday life.<br>(2) Encourage and recommend formal mindfulness practice 2-3 times a week.

(15 minutes).

Appendix 2: Mindfulness Intervention Protocols

The mindfulness intervention program comprised the following ten specific practices. Each session was guided by a trained instructor to ensure adherence to the protocol.

1. Raisin Eating Exercise

- **Description:** Each participant was given a single raisin. The exercise **required** participants to focus their full attention on experiencing the raisin's appearance, texture, smell, and taste, as well as the entire process of chewing and swallowing. The goal **was** to shift attention away from habitual, automatic eating behaviors toward mindful awareness of the present moment.
- **Key Components:** Observation, tactile experience, olfactory experience, oral experience, awareness during chewing, and awareness during swallowing.

2. Body Scan Practice

- **Description:** This practice **involved** slowly and systematically directing attention to different parts of the body (e.g., toes, legs, abdomen, chest, shoulders, and head). Participants **were instructed** to notice sensations such as tension, warmth, numbness, pain, or relaxation without judgment or attempts to change them, simply observing them as they were.
- **Key Components:** Attention guidance, awareness of sensations, acceptance and relaxation, and integrative awareness.

3. Mindfulness Stretching

- **Description:** During gentle stretching or movement, participants **maintained** full awareness of their body posture, the sensations of muscular stretching, breathing rhythm, and internal emotional changes, rather than performing movements mechanically. The essence **lay** in "moving with awareness," allowing every stretch and breath to become an object of mindfulness.
- **Key Components:** Body awareness, gentle stretching, focused experience, acceptance, and relaxation.

4. Street Encounter Exercise

- Within the campus environment, participants are encouraged to initiate brief interactions with strangers (such as asking for directions, greeting, small talk,

or offering help), followed by a reflection phase in which they discuss their emotional experiences, social perceptions, and behavioral responses. The process mainly includes: preparation and establishment of psychological safety, experiential interaction, sharing and reflection.

5. Three-Step Dissociation Exercise

- Through hierarchical psychological "step back observation", individuals are transformed from "re-experiencers" (first-person perspective) to "bystanders" (third-person perspective), thereby weakening the original emotional impact. Understand that "I am not my emotion; I am merely an observer of it." This enables participants to distinguish between "me" and "my emotion memories", achieving the reorganization of emotions and the integration of psychology. The program includes: re-entering (recalling) the situation, retreating to "outside the frame", and taking another step back (" observer ").

6. Idea Meditation Practice

- During meditation, participants do not suppress or follow their thoughts but observe them as they arise, change, and fade away. When a thought appears, one simply notes, “This is a thought,” without judging or analyzing it, allowing it to naturally come and go. The process mainly includes: calming the body and mind, open awareness, labeling thoughts, maintaining neutral observation, returning to the present moment.

7. Psychological Education on Experience Avoidance/Acceptance

- This session introduces the concept of “experience avoidance/acceptance,” helping participants understand that psychological pain is not an enemy but an experience that can be gently observed and held with awareness and compassion.

8. Mindfulness Meditation on Difficulties

- **Description:** In this meditation, participants intentionally **brought** attention to a present or past experience that **evoked** pain, discomfort, or tension. They **observed** these “difficult experiences” across physical, emotional, and cognitive levels with an open, nonjudgmental, and compassionate attitude to

promote acceptance, release, and psychological integration.

- **Key Components:** Focusing on the difficulty, body awareness, awareness of emotions and thoughts, compassion, and acceptance.

9. Steps of Emotional Acceptance

- **Description:** Participants **learned** that emotions are not something to be passively endured but can be engaged with actively, mindfully, and gently to achieve emotional acceptance.
- **Key Components:** Awareness, allowing, exploring, compassionate presence, integration, and letting go.

10. Metaphor of the Garden Meditation Practice

- **Description:** The inner world **was visualized** as a garden, which might contain beautiful flowers as well as weeds, stones, or barren patches. Through mindful observation, participants **learned** to gently care for their inner garden rather than judge, neglect, or forcefully change it.
- **Key Components:** Imagining the garden, exploring the garden, tending the garden, feeling change and gratitude, and returning to the present.
